# Challenges and Opportunities for Advancing Work on Climate Change and Public Health

**DOI:** 10.3390/ijerph121215010

**Published:** 2015-12-09

**Authors:** Solange Gould, Linda Rudolph

**Affiliations:** 1 School of Public Health, University of California, Berkeley, 50 University Hall #7360, Berkeley, CA 94720, USA; 2 Center for Climate Change and Health, Public Health Institute, 555 12th St. 10th Floor, Oakland, CA 94607, USA; linda.rudolph@phi.org

**Keywords:** climate change, public health, health equity, qualitative research, public health practice, intersectoral collaboration

## Abstract

Climate change poses a major threat to public health. Strategies that address climate change have considerable potential to benefit health and decrease health inequities, yet public health engagement at the intersection of public health, equity, and climate change has been limited. This research seeks to understand the barriers to and opportunities for advancing work at this nexus. We conducted semi-structured in-depth interviews (*N* = 113) with public health and climate change professionals and thematic analysis. Barriers to public health engagement in addressing climate change include individual perceptions that climate change is not urgent or solvable and insufficient understanding of climate change’s health impacts and programmatic connections. Institutional barriers include a lack of public health capacity, authority, and leadership; a narrow framework for public health practice that limits work on the root causes of climate change and health; and compartmentalization within and across sectors. Opportunities include integrating climate change into current public health practice; providing inter-sectoral support for climate solutions with health co-benefits; and using a health frame to engage and mobilize communities. Efforts to increase public health sector engagement should focus on education and communications, building leadership and funding, and increasing work on the shared root causes of climate change and health inequities.

## 1. Introduction

This research examines why the public health sector in California is not more engaged with climate change work given current and impending threats to health, and the potential for climate change solutions to improve health outcomes and decrease health inequities (the unfair and avoidable differences in health status between populations). The purpose of this research is to inform efforts to increase public health work on climate change, strengthen partnerships between public health and climate change efforts, and formulate strategies that address the health impacts of climate change and health opportunities in climate change policies and planning.

Climate change already endangers human health and well-being in numerous ways [1,2]. Climate change threatens to disrupt the life support systems on which humans depend (food, air, water, shelter, security), and therefore, in concert with other global environmental changes, threatens our survival. Climate change also disproportionately impacts poor and marginalized populations [3,4], thereby increasing health inequities. Climate change policies and strategies could have significant and immediate beneficial effects on public health and health equity, or “co-benefits”, through improving social determinants of health across many sectors [5,6,7]. The social determinants of health are the conditions in which people are born, grow up, live, work, and age. These circumstances are shaped by the distribution of money, power, and resources at global, national, regional, and local levels, and are mostly responsible for health inequities [8]. Through their effect on social determinants of health, some climate change strategies can *increase* the environmental, economic, and health burdens on communities already bearing the burden of cumulative environmental exposures, discrimination, poor health, and poverty [3].

Public health involvement in addressing climate change is crucial. Public health actions can do much to protect people from some of the health effects of climate change, with early action providing the largest health benefits [9,10]. The health sector can play a vital role in helping the public and policy makers understand the magnitude of climate change effects on human health, and opportunities for health and health equity promotion in climate actions.

Many major public health organizations and leaders recognize climate change as an urgent public health issue [11,12,13,14,15,16,17,18] and have argued that there is an immediate need to develop a national public health workforce that can research and address the effects of climate change on human health [19,20,21]. Some state and local public health departments are conducting projects on climate adaptation, through funding from the Centers for Disease Control and Prevention [22,23,24,25,26], and many public health departments and programs are doing work that can achieve greenhouse (GHG) reductions, which is an important aspect of preventing further climate change (e.g., promoting physical activity through walking and biking) [27].

Yet public health engagement on climate change has been limited in light of the severity of the risks of climate change, and the magnitude of the opportunities climate change solutions present for health. One assessment of local agencies found that the gap between the demand for health and climate interventions and the resources available to health departments was greater than in any other sector [28]. Prior surveys exploring U.S. public health officials’ perceptions and capacity for action on climate change [27,29,30,31,32,33,34] indicate that public health practitioners are aware of climate change and its effect on their jurisdiction, but report inadequate knowledge, information, planning, funding, resources, and workforce capacity to address this issue. To understand why these barriers persist and identify ways to promote engagement, we sought to understand the differences between public health practitioners currently working on climate change and those who are not. We also sought to understand the perspective of those working on climate change in other sectors on the potential for strengthening inter-sectoral collaboration (for this paper, inter-sectoral collaboration refers to the coordinated efforts of two or more sectors within government to improve outcomes, including working horizontally and vertically across different levels of government (local, regional, state, federal) [35].

## 2. Experimental Section

### 2.1. Research Design, Sampling, and Recruitment

We conducted semi-structured in-depth interviews with three categories of respondents from governmental and non-governmental organizations, described in Table 1.

**Table 1:** Categories and definitions of interviewees.

Interviewee Type	Definition	N
PH-engaged	Public health professionals who report working on climate change and health, and articulate explicit relationships between their work and climate change without prompting.	40
PH non-engaged	Public health professionals who do not report working on climate change, and do not articulate connections between their work and climate change without prompting.	31
Non-PH	People whose work focuses on climate change, and who do not primarily identify as public health professionals.	42

We began with a purposive sample of “PH engaged” in climate change and “Non-PH” professionals we knew through practice or published literature who were working on climate change as a primary focus of their work. Examples of “PH engaged” participants’ activities include research on the public health impacts of climate change, participation in climate change policy-making and planning to provide a health lens, and explicit integration of climate change co-benefits in chronic disease prevention activities. Through snowball sampling, we identified “PH non-engaged” colleagues working on areas with a potential public health relationship to climate change (e.g., physical activity nutrition, asthma), and enlarged our sample for all categories. Individuals were invited to participate with a detailed email, follow-up emails and phone calls leading to a participation rate of 84%.

We developed, piloted, and revised semi-structured interview guides for each category of participants (see Appendix Information for interview guides). We conducted 113 interviews of one to one and a half hours in-person or via telephone from May through September 2013. Participants were mainly from California, with a smaller sample of national public health leaders engaged in climate change. We asked interviewees about their knowledge, attitudes, and activities regarding climate change and health, strategies to address climate change, and challenges to and opportunities for greater engagement in work at that nexus. For the public health interviewees, our final determination of participant category was based on whether they articulated their work as connected to climate change without prompting during the interview. Table 2 further describes participants.

**Table 2:** Participant attributes by interviewee type.

Participant Attributes (*N* = 113)	% of Total (*N*)	PH Engaged % (*N*)	PH Non-Engaged % (*N*)	Non-PH % (*N*)
**Total interviewee type**		35 (40)	27 (31)	31 (42)
**Location**				
California	90 (102)	77.5 (31)	100 (31)	95.2 (40)
* San Francisco Bay Area and Northern California	44 (50)	58.1 (18)	54.8 (17)	37.5 (15)
* Sacramento area	18 (20)	9.6 (3)	12.9 (4)	32.5 (13)
* San Joaquin Valley	11 (12)	0 (0)	22.6 (7)	12.5 (5)
* Los Angeles and Southern California	18 (20)	32.3 (10)	9.7 (3)	17.5 (7)
Other States	10 (11)	22.5 (9)	0 (0)	4.8 (2)
**Organization Type**				
Academic	8.8 (10)	20 (8)	0 (0)	4.8 (2)
Governmental	34.5 (39)	40 (16)	54.8 (17)	14.3 (6)
Non-governmental Organization	56.6 (64)	40 (16)	45.2 (14)	81.0 (34)
**Position in organization**				
Non-executive	47.8 (54)	60 (24)	22.6 (7)	54.8 (23)
Executive or Director	52.2 (59)	40 (16)	77.4 (24)	45.2 (19)
**Gender**				
Female	57.5 (65)	62.5 (25)	51.6 (16)	57.1 (24)
Male	42.5 (48)	37.5 (15)	48.4 (15)	42.9 (18)

* California regional percentages based on California totals.

### 2.2. Analysis of Data

Transcribed interviews, notes, and descriptors were imported into Dedoose [36] qualitative data analysis software. First, the research team sorted data into broad “big bucket” code topics derived directly from the survey instrument. To ensure coding validity, three coders independently coded the same transcripts, compared them, discussed discrepancies in coding, and refined the coding scheme until reaching a high level of agreement on code application. The authors then conducted additional sub-coding, in-depth inductive and deductive thematic analysis, and analysis of patterns within and across participants groups and organization types.

Broad code topics for the analysis presented here included: (1) public health role in climate change, (2) barriers to public health participation, (3) opportunities supporting public health engagement, and (4) recommendations. Examples of sub-codes included urgency, leadership, politics, authority, siloed thinking and approach, public health as messenger, and support other sectors.

## 3. Results

We present results of two main areas of analysis: Barriers to effective public health engagement, and opportunities for advancing work at the nexus of climate change and public health. Some barriers operate at a conceptual individual-level, while others operate within the institution of public health or are structural (operating across multiple interconnected systems and sectors). We first present the findings on barriers from the two categories of public health interviewees (“PH-engaged” and “PH non-engaged”), and the “Non-PH” perceived barriers at the end. Opportunities to shift public health practice towards stronger engagement on climate change issues include integrating climate change into current public health practice; providing support for climate solutions with health co-benefits; communicating potential health and health equity impacts of climate change and climate actions; and engaging and mobilizing impacted communities and public health professionals.

### 3.1. Barriers to Engagement

#### 3.1.1. Public Health Perspectives on Individual-Level Constraints

##### *Belief that Climate Change is not Urgent, Immediate, or a Priority* 

The “PH engaged” respondents were, not surprisingly, highly aware of the health risks of climate change and the urgency of response. A number of these respondents from public health organizations recognized as leaders in climate change work did not feel it was a priority of their organization. The primary motivation for many “PH engaged” respondents’ engagement in climate change work is that those activities serve as a platform to address larger community health needs.

While there was interest and concern about climate change among “PH non-engaged” professionals, this group expressed less interest, urgency, or recognition of the breadth and currency of climate impacts on health, or that interventions are required now to prevent more severe future impacts. One respondent noted that the health impacts of climate change “*seem like a reach.”* Many of these respondents see the impacts as too distant in time and place to be of concern, “*a pervasive perception that climate change will happen in the future”,* although several acknowledged the need for public health response to extreme weather events. Almost all respondents—including those engaged with climate change—believe there is a lack of tangibility or immediacy to the issue; that makes it too abstract for people to engage with. For those “PH non-engaged” respondents that did perceive climate change as an immediate and urgent threat, it was not a priority for their organization or agency.

“PH non-engaged” interviewees expressed skepticism that climate change was a pressing issue for the communities they serve, relative to gun violence, unemployment, or housing issues. One respondent pointed out: *“We can’t expect people to think about the future if they don’t have their immediate needs met.”* The exception to this was “PH non-engaged” respondents working in environmental justice organizations, who highlighted the importance of environmental protection—including climate change—for the communities they represent:

“In our community, there is a deep tie to the environment that is so dismissed: Latinos care about the environment because of the environment, not just for self-interest…People feel a strong tie with the land…We came from agrarian societies, and voters would support policies that protect water, trees, and land, even with costs associated. They understand the psychological benefits of preserving the earth, as well as the health benefits…That view is not well understood in the larger equity movement.”

##### *Insufficient Understanding of Impacts, Connections, and Solutions* 

Lack of self-efficacy emerged as a theme in several dimensions. The “PH non-engaged” respondents felt they lacked adequate information or expertise on health impacts of climate change, the health impacts of climate change planning and policies, and the connections between current public health work and climate change:

“There is a lack of knowledge that climate change has impact across sectors…In public health, people work on one narrow issue and folks might have a hard time making the links between their public health work and climate change.”

“PH non-engaged” participants demonstrated low awareness of connections between climate change and their current work, as exemplified by these statements by three participants:

*“We do air quality projects… I’m very familiar with the concept of co-benefits but have never thought about it with regard to climate change.”* And, *“It’s uncanny how connected our work is to climate change. It’s almost coincidental. But I wouldn’t necessarily link Safe Routes to School or physical activity to climate change.”* Finally, *“WIC would be active in obesity, nutrition, social and environmental determinants of nutrition, but I don’t think of it as a climate change issue. The climate change issue is what happens when the food supply gets disrupted. That is emergency preparedness.”*

Other “PH non-engaged” respondents were aware of the connections between their work and climate change, but expressed distinct unease at making these links explicit with their peers, community stakeholders, or decision-makers because they are uncertain about the local impacts of climate change and how it will affect the communities they work with, One “PH non-engaged” respondent said, *“I know it’s a big issue, but I lack data to talk to community and decision-makers at the appropriate scale.”* In fact, both groups of public health respondents cited a need for better down-scaling of local climate change impacts, research on community impacts, and methods to forecast local health impacts based on down-scaled climate data. Significantly, many “PH engaged” respondents also did not feel they were experts, understood only one area such as emergency preparedness, and did not think systemically or about multiple impacts.

Respondents from both public health groups expressed frustration at the difficulty of identifying specific meaningful solutions that match the scale and complexity of problem. Even respondents who understand that their own work may help to address climate change expressed skepticism about the effectiveness of their role: *“The work of climate change gives me an overwhelmed feeling. I have a cynical feeling, like how much could it help, promoting Safe Routes to Schools?”*

#### 3.1.2. Public Health Perspectives on Institutional and Structural-Level Constraints

Public health respondents from both groups identified myriad institutional and structural constraints that make efforts to engage in climate work challenging. Structures refer to the inter-institutional arrangements and interactions (such as laws, policies, and standard operating procedures) whose joint operation produce health and equity outcomes [37]. The institutional constraints operate within public health agencies and the public health sector, which together operate as the public health institution, while structural barriers operate across multiple interconnected systems and sectors outside of public health.

##### *Lack of Capacity in Public Health Infrastructure, Funding, and Workforce* 

Respondents from both public health groups said that adding climate change to existing public health priorities seems overwhelming without infrastructure, resources, and workforce capacity, especially given significant recent budget cuts. Two “PH engaged” respondents explained:

“Public health has taken a huge hit in staffing with most public health departments at 40%–60% of their staffing levels prior to ten years ago, but have more on their plates. The field of public health keeps growing, and we need to prioritize what public health agencies should be doing. Protecting from climate change is one of those things…”

“The public health workforce is being eroded rapidly…We will be down staggering numbers. They will need to be paid a living wage and have the tools to work on public health and climate change and be well equipped. It takes more than a guidebook, it takes training, monitoring systems, doing outreach and trainings, and that all takes dollars.”

The extent to which public health funding is largely through categorical programs with limited and tightly defined scopes of work exacerbates this view. One “PH engaged” said:

“You’re trying to do climate change work on top of everything else you’re doing. There is no funding stream, no time set aside. I try to carve time out to focus on it. But you have all these other things that you’re getting paid to do.”

Another agreed:

“We’re compartmentalized, and have mandates, missions, and activities that we do every day… feeling stressed in getting them accomplished. If we tell people they need to add climate change, there is a lot of resistance.”

Climate change cuts across many programmatic areas, yet respondents in both public health groups bemoaned the perception of program administrators that unless climate change is specifically mentioned in a program’s scope of work, funding cannot be used to address it, even if the grant or contract language might allow it.

Many “PH engaged” expressed frustration with the difficulty of raising funds for work on climate change and public health, and how this limited interdisciplinary collaboration:

“The (X) Foundation, which gives incredible amounts of money to public health, specifically doesn’t fund environmental problems. That decision holds implications on the ability to be involved in issues like climate change. That really reinforces silos between public health and other disciplines, and within public health.”

##### *Formal and Informal Authority* 

Numerous respondents in both public health groups spoke about the importance of legal mandates to support public health engagement in climate change. Jurisdiction for implementation of climate change mitigation and adaptation policies varies amongst states that have policies in place, but are generally under the purview of environmental organizations such as the EPA, air boards, fire, water, and conservation agencies. Public health agencies are usually mandated to respond to emergent public health threats such as those caused by climate-related natural disasters and disease outbreaks, as well as limited occupational regulation (e.g., for heat risk). Few public health agencies have any explicit climate change-related mandates. A “PH engaged” gave this example:

“The overall direction and mandate in public health is missing the climate change component. How do you make heat-related illness a reportable disease to elevate it to other public health priorities like sexually transmitted infections?”

Another “PH engaged” said:

“Public health is struggling to see how it fits in. From an agency perspective, people think it’s relevant, but are constrained by the fact that they don’t have jurisdictional authority to do something about it. Addressing climate change would be mitigating greenhouse gases, but there is no jurisdictional authority related to that at all. They may feel that their hands are tied.”

While public health respondents said that they lack formal authority over climate change’s health impacts or drivers, respondents from all groups believed that the public health sector has a lot of informal authority amongst communities and decision-makers. They believe that public health professionals could use this informal authority to provide greater leadership and possibly even move towards formal mandates for public health to address climate change.

##### *Lack of Leadership: Risk Aversion and Politicization of Climate Change* 

However, across all groups there was a high level of consensus and frustration that public health leadership on climate change is sorely lacking. Strengthening leadership amongst the executive leaders *of mitigating climate change, just raising their voices more in the public.”* who define priorities and allocate resources was identified as a crucial step. “PH engaged” participants voiced frustration with the absence of public health agency leadership and action on climate change:

“Public health should be speaking out more on the importance of addressing climate change”

When asked why this was not happening, participants linked this to politicization, lack of higher-level institutional support, overt pressure to not act, and fear of repercussions in a risk-averse culture.One governmental “PH engaged” said working on climate change depends on “*if they have the leadership, vision, or leeway to get into things elected officials are going to slap them down for.”*

Those “PH non-engaged” who saw connections in their work to climate change expressed reluctance to make the links explicit based on perceived political constraints. In jurisdictions where leaders dispute that climate change is real or human-caused, there is fear about even mentioning the words “climate change”, as expressed by a “PH non-engaged”:

“In a public health department like this, the legislation controls your budget, and if there are legislators who feel in any way threatened by interventions, you could find yourself not getting funding for different things.”

In addition, participants from all groups believe the lack of public health leadership on climate change is exacerbated by a risk-averse culture in public health. One “PH engaged” governmental employee noted:

“Some other parts of the government are less risk averse, but ours is more so… Their whole thing is that ‘we don’t want any surprises and everything goes through us’. Anything that causes anyone to be upset, they shy away from it.”

Another “PH engaged” respondent said:

“They need to know the Board of Supervisors (BOS) won’t fire them if they speak out on climate change…. Not until people are at the end of their careers will they say what needs to be said.”

Significantly, public health respondents from both groups indicated that the politicization of climate change and the risk aversion limiting public health involvement impacts their ability to fulfill basic roles, such as stating the likely health effects of policies being considered or the health conditions in their community that may be related to climate change.

##### *Narrow Framework Limits Work on Root Causes of Climate and Health* 

Because many of the actions to address climate change include efforts to address systems change (e.g., transportation), respondents in both public health groups voiced a need for stronger public health practice on upstream prevention and the social determinants of health, such as food systems, active transportation, healthy housing, and the built environment, as well as more training and resources for that work. A “PH non-engaged” respondent explained:

“Most of what we do as a public health department is service delivery. Prevention—which is what climate change work is—is a very tiny part of what we do.”

A “PH engaged” participant agreed:

“So many public health departments are still entrenched in how funding is coming, silos, disease conditions, and individual behavior change. Those local health departments that are at least looking at neighborhood conditions will be better off. Climate change is still pretty far out.”

Recognition that climate change is complex and requires a sustained commitment led respondents to express frustration at a *“reactive”* public health practice dominated by the *“tyranny of the urgent”*. “PH engaged” respondents stressed that in order to work on climate change, public health will have to shift its resources and attention from service provision to systems changes:

“Other things will have to go and be shifted to someone else’s responsibility so that public health can do its work at the broad population level…and focus on the macro-level changes that are needed.”

A “PH engaged” respondent stressed that the root causes of climate change should be the focus, even beyond mid-stream environmental exposures:

“The focus on the air pollution benefits is myopic. That’s another challenge for public health: Even within the EPA, everything is organized according to a disease outcome or a health issue. Climate is a large problem that requires systems thinking. If we limit the conversation only to (air pollution), that’s short sighted without thinking about underlying issues.”

##### *Compartmentalization within and across Sectors* 

In order to work on systems changes, public health will need to work effectively across public health programs, and across other sectors. Compartmentalization and lack of coordination across funding streams prevents work on cross-cutting issues like climate change and health. Respondents from all groups note that the silos become more challenging when working across sectors, given organizational barriers and differences in language, approach, and priorities:

“We don’t understand each other at all. We’re in different disciplines; don’t have knowledge of their expertise, different language, and different emphasis on what’s important. It takes twice as much work; you have to get a rudimentary understanding of what they’re doing. Some ways to get over that is willingness to spend a little more time making sure people understand each other.”

One “PH engaged” government worker described territorial conflicts over climate change:

“There’s still a sense that public health shouldn’t be in the room because things are not a health initiative. But all public agencies should be responsible to the public and part of that is keeping them safe and healthy. There are turf issues, especially with infrastructure agencies, transit authority, or pollution-planning.”

Another “PH engaged” participant described:

“Air pollution control district and planning are strong partners, but we still tend to get siloed, and they still think climate change is theirs. They all talk ‘health’, but don’t invite health to the table. (X University) organized a state-wide conference, and health was a major topic on the agenda, but they never invited (local health department) to the conference.”

#### 3.1.3. Non-Public Health Perspectives on Barriers to Public Health Engagement

In contrast, the “Non-PH” respondents identified very few barriers, and could identify numerous immediate roles for public health (described below in “3.2. Opportunities for Engagement”). However, “Non-PH” participants voiced frustration with the absence of public health agency leadership, action, and participation in contentious climate change policy-making:

“I don’t know why public health feels that they are not allowed to do that. (A few public health leaders) are willing to stick their necks out, take political risks, and state what will happen to health with various policies.”

This group of respondents believed that the compartmentalization and lack of coordination across funding streams prevents work on cross-cutting issues like climate change and health, and warned against dedicated categorical funding for climate change. One said:

“One of the most disheartening times in the movement was when people started to just fund climate, thinking it was its own thing, but it’s connected to every issue we work on. If you’re working on clean air, on food, you’re working on climate.”

Related to this, “Non-PH” participants described this as an important barrier, because:

“*The siloed mentality also means we don’t get the big picture… this is all inter-related.”*

Another “Non-PH” participant agreed:

“Part of the issue is a lack of integration and a lack of systems thinking. We look one piece at a time, but this affects us in a multitude of ways and we need to bring people together to solve these problems. We always solve one thing and create another problem, like with biofuels. There are water issues, rising food prices, even immigration problems that arise from making decisions in isolation.”

### 3.2. Opportunities for Engagement

The “PH engaged” and “Non-PH” participants identified similar opportunities for public health sector engagement through integration of climate change into existing programs, engagement in climate action planning and policy, support for climate solutions with health co-benefits; and communicating with, engaging and mobilizing impacted communities and other public health professionals. “PH non-engaged” participants expressed a desire for further training and education on climate change and public health, and how they might become engaged.

#### 3.2.1. Integrate Climate Change into Current Public Health Practice

Both public health participant groups expressed doubt that there would be significant new funding for public health agencies to work on climate change, suggesting a strategy to integrate climate change into existing programs where feasible. To promote greater engagement respondents advised:

“…first help people see that they already are influencing issues around climate change with what they do every day. Just tweaking and finessing it rather than completely changing their workload.”

Examples provided included integration of climate projections into accreditation-required community health assessments and public health emergency preparedness, home visitation program referrals for energy efficiency improvements, articulation of climate co-benefits in chronic disease prevention programs (e.g., active transportation), and inclusion of climate-related health outcomes in existing disease surveillance programs. Respondents cited the need for greater funding flexibility and workforce capacity building to implement these suggestions.

“PH non-engaged” and “Non-PH” respondents asked for more information on the range of health and equity impacts of climate change and climate solutions—including downscaled local health impacts—and how these connect to current work. A “PH non-engaged” respondent observed:

“We are involved with work on air quality, safe neighborhoods, reduction of traffic, water quality, biking paths, walkable communities, and obesity prevention (through community gardens, famers markets, and city planning). I do not make the links explicit currently, but would be interesting in doing so with guidance on ‘how to’”.

Some “PH engaged” respondents noted that adequate evidence for action already exists, and that the real need is for greater dissemination of existing knowledge:

“The public health sector needs to get their research out there. Good work has been done, but it has not effectively been shared with the public…both at community and institution level. This would provide opportunities for advocacy and help put pressure on legislators to introduce stronger legislation with more information on public health.”

#### 3.2.2. Provide Active Inter-Sectoral Support for Climate Solutions with Health Co-Benefits

“PH engaged” respondents explained that most climate change strategies and solutions work on social determinants of health, and that public health’s presence in this inter-sectoral work would improve health inequities and resilience across many outcomes. Climate change work could also be a vehicle for getting out of the silos and limited framework that public health is perceived as being constrained by, thereby improving public health practice broadly. A “PH engaged” said:

“There’s a lot of research supporting that active transit and affordable housing will have a climate change impact. It’s a multi-step connection, but people don’t often make the connection in lectures to elected officials or presentations at Commission. They sometimes connect the dots, but they may just be listed as a bullet on why active transportation is good.”

A “Non-PH” participant agreed:

“With health and adaptation, it’s still complicated for a planner to understand. We understand extreme heat events, but I don’t have a background in terms of thresholds and what numbers matter. It’s still hard for me to understand what the community impact will be. I couldn’t go the next step and say the risk for my community in terms of health is this. Public health can make that data more accessible to planners like me.”

Respondents from all groups identified climate change actions with health co-benefits, such as active transportation, wildfire prevention, or agricultural land conservation, and believed that more active public health support could enhance decision-maker support for these policies. Some suggest building on Health in All Policies and Health Impact Assessment practice, and bringing a health equity lens to climate change discussions. “PH engaged” felt strongly that both climate change mitigation and adaptation must be addressed, but several believed that mitigation might offer greater opportunity to address the social determinants of health.

“Non-PH” respondents—especially non-governmental organization staff—felt the public health sector should use its special authority and *“show up”* for climate change decision-making.They emphasized that they were ready, eager, and willing to incorporate health impacts and co-benefits into their climate change work, and partner with public health to bring public health implications of climate change work into public debate and decision-making spheres.

#### 3.2.3. Engage and Mobilize Health Professionals and Impacted Communities with a Health Frame

Respondents engaged with climate change from within public health and non-public health said that there was a need for a larger movement on climate change. Across groups, but particularly in the “Non-PH” group, respondents felt that *“the public health sector has a lead role to play”* in facilitating this magnitude of change. As a first step, several respondents suggested that public health needed mobilization from within the field. They suggested a coordinated, funded strategy that convenes the public health sector, aligns their various work to address climate change, and facilitates a larger health movement for climate change.

Foundations were seen as having a significant role in facilitating this movement by coordinating funding streams and creating a collective impact approach for work at the intersection of climate and health. Some respondents referenced the sense of urgency, strategy, and unity of purpose when foundations set priorities and aligned funding streams around place-based health and obesity prevention; seemingly unrelated public health activities across a range of issues now appeared to be integral parts of a strategic movement with a purpose and vision. The foundations provided the leadership, and public health leadership followed suit.

Next, “Non-PH” respondents held that “*There is a vacuum in the government in communicating with the public, and public health should claim that as its area.”* They would like to see health-framed communications about climate change and its health impacts, and strongly believed that the public health sector has the credibility and connection to communities that could be used to mobilize public support for health-protective action on climate change. One respondent who had experience framing climate change as a public health issue in political campaigns noted that public health:

“*…was the motherhood & apple pie of that (political campaign). Our opponents couldn’t criticize or devalue them as a messenger. They were a messenger beyond reproach, with a message beyond reproach”.*

“Non-PH” participants wanted public health to use this “*heft*” more to change public discourse around climate change:

“There is a lot of messaging that the public health community could do, to create an environment where policy can thrive. We have pushback from conservative movements. People who are wavering and on the fence, don’t take time to read science, they can hear a public health voice more than a planner or politician.”

However, some “Non-PH” respondents noted the need for a more coherent public health communications strategy on climate change, grounded in better research and lessons from prior successful social norms change campaigns *“that have moved a whole population, be it Mothers Against Drunk Driving or Anti-Tobacco, and that really changed the social fabric and the way people think about things and what they’re willing to do.”* Theserespondents suggested that public health could provide a unifying frame linking climate change, health, environmental justice, social justice, and equity, and supporting an integrated approach. “*There is good work happening in silos, but the community needs a more cohesive vision to move forward and health might provide that cohesion.”* Some respondents felt that public health messaging could ground “climate change” framing, in that: *“Public health makes climate change real for these communities.”*

Finally, respondents across groups believed that public health could partner more strategically with climate change organizers to engage, partner with, and mobilize communities most impacted by climate change. Many noted that public health agencies already work with these communities, and that public health workers can work with community members to identify and define climate and health problems, and ensure that climate solutions do not negatively impact disadvantaged communities. One climate change organizer stated, *“Human rights and health have narrative power…It’s a great litmus test for most policy proposals.”* Another noted: “*Every municipality in the city is working on climate action planning. We can be at every one of those… and health can get us there, connect grassroots groups to each other so there is something to mobilize for. We haven’t had that meta-frame.”*

## 4. Discussion

Our research adds a more nuanced examination of the barriers and opportunities for increasing public health sector work addressing climate change. Strengths include an in-depth qualitative assessment and the inclusion of groups with different perspectives on the issue, particularly those who are not engaged with climate change. The research is limited by the fact that the sample was not representative of public health professionals, either in level of engagement on climate change or geographically. The predominance of California respondents could skew results to reflect the state’s broad support for aggressive action on climate change. Within California, 58% of the respondents were from the politically progressive San Francisco Bay Area, further skewing results to reflect support for action on climate change. Our sample did not include any public health professionals from other states who are not engaged with climate change; follow up studies should investigate involvement with climate change in this target group. It is also worth noting that the majority of the public health professionals not engaged in climate change were in executive positions; further research should investigate this subgroup since many interviewees believed that organizational leaders were a crucial group to engage. Finally, further studies could investigate public health academics who are not engaged in climate change. As thought leaders and trainers for the next generation of public health professionals, they are an important subgroup to engage.

This research suggests that there are many opportunities for the public health sector to become more engaged in work that addresses climate change, but that broader engagement will progress slowly without more explicit strategies to improve public health sector understanding of the issue, address institutional barriers, build leadership, and shift public health practice and funding towards root causes of climate change and poor health.

### 4.1. Education and Communications for the Public Health Sector

In prior surveys of public health professionals, the majority of participants recognized that climate change impacts health. Many of the public health professionals we interviewed do not know or understand much about climate change and health nor believe they have adequate information to engage on the issue. Participants also did not appear to appreciate the interconnections between health, inequities, and climate change impacts and opportunities [38]. Although our research preceded the last National Climate Assessment and the release of the IPCC Fifth Assessment Report, there has been a growing body of evidence that climate change will affect health since the late 1980s, and more recent literature on the health co-benefits of climate action. A concerted effort is clearly needed to reach public health practitioners with this information in a way that fosters greater engagement and self-efficacy. More specific forecasting of the local health impacts of climate change may reduce the perception that climate change is so distant in time and place as not to pose any urgent challenge to public health.

Respondents from outside of the public health sector strongly articulated the potential value of the public health voice in communications to help catalyze public opinion to support more robust climate action. Because health education and communications are a routine component of virtually all public health programs, support for integration of climate change into public health messages could serve as a relatively easy first step to increased engagement.

### 4.2. Target Public Health Institutional Barriers

Our research indicates the persistence of deficits in funding, leadership, knowledge, and workforce capacity found in previous surveys [27]. Some of these persistent deficits in public health institutional capacity may be attributable to barriers from within the field of public health (e.g., insular practice, conflict aversion, or siloed training), while others are likely shaped by institutional constraints from outside of public health.

For example, the political controversy about the science of climate change appears to significantly influence the willingness of public health professionals to speak publicly about climate change as a significant public health issue. Even among respondents who clearly understood the issue, felt the urgency, and saw relationships between their work and climate change, few felt comfortable making the links explicit and were reluctant to take public leadership on the issue. Given the extent of local and state focus on climate change in California, however, the fact that so many of our respondents still expressed hesitancy about talking about climate change is notable.

Public health staff’s mission is to protect and promote the public’s health. Yet they are aware of and fearful about the potential consequences of being vocal about climate change when those with power over budgets and jobs disavow it. It is unknown whether increased awareness of the general public’s level of concern and desire for government action on climate change [39] and the view of those in other sectors that health professionals can play a critical role on this issue could counter the impacts of politicization. Public health has a history of action on other politicized health issues, such as HIV/AIDS, needle exchange, and reproductive health, largely in the context of social movements around those issues. This data indicates that when governmental public health staff are constrained due to politicization, strong partnerships with non-governmental organizations and “inside-outside” strategies are needed to advance climate change and public health nexus work.

### 4.3. Build Leadership

Effective leadership will be required to significantly shift public health practice to fully address climate change. Strong leadership could leverage formal and informal authority to educate the public and inform policy even in the absence of statutory mandates or significant new funding. Not every local health department or health organization will have charismatic leaders; training and support are needed to cultivate and develop the leadership necessary to face a challenge of this magnitude.

California and Federal climate change policies cite the health benefits of such policies, but often do not provide the formal authority, leadership, requirements to work with public health, or funding to public health agencies in creation or implementation of those policies. Future research should investigate effective use of informal authority, and creative use of legal authority avenues that now exist. For example, the National Environmental Policy Act and the California Environmental Quality Act (NEPA/CEQA) require many public agencies to analyze and disclose potentially significant environmental effects of agency actions, including effects on human health. The environmental impact assessment (EIA) required under these laws may be an opportunity for improving integration of human health concerns within the EIA process for climate-specific planning [40], and partnering with public agencies that do have authority to support and improve health.

### 4.4. Work on Root Causes of Climate Change and Poor Health

The way that agencies and programs are funded and organized within public health presents a significant barrier because addressing climate change requires work on multiple systems that both cause climate change and public health problems. Unfortunately due to norms of practice within the field and bureaucratic and political constraints from larger systems, work on climate change within health tends towards a narrow framework for practice, limited capacity due to a decimated public health infrastructure, funding, and workforce expertise, lack of formal power and informal leadership, and compartmentalization within public health and in most other governmental agencies necessary for effective partnership in forming systemic solutions.

Work that addresses the determinants of both health inequities and climate change provides perhaps the best opportunity to improve those systems that cause climate change, poor health, and inequities through myriad pathways. Failure to make explicit the links between community health promotion, health equity, and climate change mitigation and adaptation represents missed opportunities to inform and activate citizens to demand more action on climate change, and to inform decision-makers about opportunities to optimize health and equity or minimize harms. For example, while hundreds of municipalities around the nation are developing climate action plans, few are explicitly engaging or integrating health and equity, thus missing opportunities to prevent harms and optimize health and equity co-benefits [41]. The lessons we learn from work at the nexus of climate change and health are useful to understanding how to advance other work on social determinants of health. As respondents described in the barriers section, public health practice that remains focused on a biomedical model will miss those opportunities.

### 4.5. Coordinate and Shift Funding

Resource limitations have forced public health agencies into a reactive mode, responsive to one immediate health crisis after the next. Disinvestment in the public health infrastructure has thus weakened the ability of public health agencies to respond proactively to a slow-moving public health emergency such as climate change or address the determinants of climate change and health. Significant reinvestments in the public health infrastructure will be necessary in order to absorb the increasing impacts on health of climate change, and to enable conceptualization, organization, and practice that proactively optimize health and equity benefits from climate change work.

As mentioned in the results, respondents identified a number of strategic and creative ways to use existing funding. The fact that interviewees working on climate change outside of public health saw important and immediate roles for the public health sector supports the idea that inter-sectoral collaborations—for example the Health in All Policies approach—might incorporate more explicit linkages with climate change mitigation and resilience. Health and equity considerations could be integrated more explicitly into climate change program planning and policy development. Governmental and private funders could more explicitly permit or encourage integration of climate change, wherever relevant, in currently funded programs. Finally, foundations are setting the agenda for powerful movements around health, and these respondents are hungry for such a movement in public health for climate change.

Despite the challenges, our respondents identified an array of promising initiatives that provide hope that, as the magnitude of the climate change threat to health becomes ever more apparent, the public health sector will find ways to engage on the issue.

## 5. Conclusions

Climate change is a threat to human health and survival, and climate solutions represent one of the biggest opportunities for advancing health and equity. Our research identifies both significant barriers to and opportunities for public health and partners to simultaneously address climate change, health, and health inequities. We hope this information will motivate and facilitate the public health sector’s participation in the fight against climate change, and strengthen relationships with other sectors engaged in climate change planning and policy-making.

## Appendix

### A. “Public Health Engaged” Interview Tool

#### A1. Introduction

Thank you for agreeing to meet with me/us. We are working with the Public Health Institute Center for Public Health and Climate Change on a project funded by the Kresge Foundation. We see climate change as one of the greatest health and equity challenges of the 21st century, and we also see that many climate action strategies could have very positive impacts on health. The goal of this project is to find out how to make it easier for the public health community to work on climate change and strengthen partnerships between public health workers and others who are working on climate change. We also want to know how public health could help others working on climate change to bring a stronger health frame to their work. We are starting by speaking to people in public health, climate change, equity, planning, environmental justice, and other fields, to learn more about their work and get their ideas on what would be helpful.

Everything you tell me will be kept strictly confidential, unless we specifically ask for your permission to quote you. I‘ll be taking notes, but only my co-investigator and I will have access to them. You should feel free at any time to ask me questions concerning the interview or the project, or to decline to answer a question. Do you have any questions? OK, let’s get started.

It will help to define what we mean by public health and climate change up front.

We’re defining “public health” broadly to include the “upstream” or social, environmental and physical determinants of health, such as asthma from air pollution, housing conditions, education, income, access to healthy foods and transportation, *etc*. When we talk about “public health workers” or “public health organizations”, we are talking about people both inside and outside of government that are working on any aspect of trying to improve population and community health.

When we talk about the impacts of climate change on health, we’re talking about both direct impacts like extreme heat, indirect impacts like rising food prices from drought, and the health effects of various strategies to reduce greenhouse gas emissions such as decreased air pollution from higher fuel efficiency. Also, this project is focused more on the US than on international issues, even though we understand that the global impacts are very significant.

(Note: * indicates questions you can skip if pressed for time.)

#### A2. Public Health Involvement and Role

First, can you tell me a little about the work you do, especially work which you think relates directly or indirectly to climate change or co-benefits?

* You’ve talked about climate change and its links to (public health topic areas they mentioned). Are there other areas where you see key connections between climate change and public health, for example like emergency preparedness, vector-borne diseases, chronic diseases, and how do you see those links?

- What about areas like maternal and child health, infectious diseases, or reproductive health?

What are your thoughts about the role of public health and public health workers in climate change—what should the public health community—inside and outside of government public health agencies—be doing about climate change? If you had lots of resources, in an ideal world, what would public health be doing around climate change?

Do you think that we should be striving to get everyone in public health engaged in climate change, or do you think we should focus on building expertise in a smaller more focused group of public health workers, or both? Do you have any thoughts about reaching out to workers engaged in direct service – like public health nurses, or restaurant inspectors or WIC workers about climate change, and whether they could or should have a role in climate change work?

#### A3. Framing

Do you think there is a way we could use a health frame or health focus to help the public become more aware of and engaged with climate change?

*Probes*:

- What about areas like maternal and child health, infectious diseases, or reproductive health?
- Are there any health messages frames that been particularly effective in your experience, or ideas you have as to what you think could be most compelling for your co-workers?
- What about for the clients and population that you work with?

Some people—with some support from polling data—think that it’s important to talk about the health co-benefits of climate action—for example, the air pollution benefits of (AB32), but to refrain from saying the words “climate change” or “global warming”; others think it’s very important to actually say the words “climate change”. What are your thoughts about that?

#### A4. Barriers

* What are the barriers to public health participation and input into work that’s happening locally and regionally around climate change? I’d like you to think about both “upstream” or structural barriers, and more immediate barriers. Probe:

- Are there specific issues, organizational, time, resources, leadership, *etc.*, that make it hard for you to engage in climate-related work?

Do you have thoughts about why public health workers overall are not more engaged in climate change?

- Are there specific issues, organizational, time, resources, leadership, *etc.*, that make it hard for you to engage in climate-related work?
- What do you think it would take to get public health workers more engaged?
- What would be effective ways to motivate and activate public health workers on the issue of climate change?

#### A5. Opportunities

* What are some of the things that might help and support others in the public health sector become more interested or engaged with work related to climate change and health? Prompts:

- More information and understanding,
- time,
- resources,
- funding,
- leadership,
- peer support

* Who should we be targeting with that support?

Do you see any key opportunities for public health in terms of local, state, or national climate or health policy engagement?

What about key opportunities for community education and engagement?

What about opportunities for partnerships with other sectors, other organizations, or other initiatives related to climate change that public health hasn’t yet been involved in?

#### A6. Multi-Sector Planning and Decision-Making for Climate Change and Health

Have you been involved in any climate change mitigation or adaptation planning, or other climate action work locally, regionally, or at the state or national level?

- What do you see as any unique contribution or possible contribution of public health in that work?
- Did others see PH as having a unique contribution, and what was that?

Who are the other stakeholders or organizations that you think public health should be working with on climate-related issues?

#### A7. Narratives about Public Health and Climate Change

What made you first get engaged in climate change work? Can you tell me what, when, or who made you start caring about it or thinking it was an important public health issue?

What do you find most rewarding about it?

Can you tell me a story about an accomplishment or something you feel really good about in the work you are doing?

- Did others see PH as having a unique contribution, and what was that?
- Why does that story stick in your mind?
- How does that story make you feel?

What is the most disheartening aspect of your work on climate change and public health? How do you deal with that?

Are there other areas where you would like to expand your work related to climate change, climate-related impacts, or co-benefits?

#### A8. Networking

Would you mind sharing the names of others who you think would be useful to interview on this subject? We are looking for other public health workers with some awareness or interest, and who may be able to increase their agency’s engagement. *(Get contact information)*

Would you be interested in receiving information from the PHI Center for Climate Change and Health by email?

Do you have any questions for me/us? Is there anything else you’d like to share?

You’ve been so helpful; I really appreciate the time you’ve taken to talk with me today. I just want to confirm that we can list you in the acknowledgements section of our final whitepaper. Do you mind if we contact you in the future with any follow-up questions that may emerge? Thank you very much.

### B. “Public Health Non-Engaged” Interview Tool

#### B1. Introduction

Thank you for agreeing to meet with me/us. We are working with the Public Health Institute Center for Public Health and Climate Change on a project funded by the Kresge Foundation. We believe that climate change will pose a challenge to health and equity in the 21st century, and we also see that many climate action strategies could have very positive impacts on health. The goal of this project is to find out how to make it easier for the public health community to work on climate change, and strengthen partnerships between public health workers and others who are working on climate change. We also want to know how public health could help others working on climate change to bring a stronger health frame to their work. We are starting by speaking to people in public health, climate change, equity, planning, environmental justice, and other fields, to learn more about their work and get their ideas on what would be helpful.

We are going to ask you questions about climate change, but we know that it is not an issue that many people in public health know all that much about. So please don’t worry about whether or not your answers are “right”—for many of the questions there are no “right” answers. We are asking them so that we have a better understanding about where we should start when, later in our project, we develop some materials about climate change specifically for public health workers.

Everything you tell me will be kept confidential, unless we specifically ask for your permission to quote you. I’ll be taking notes, but only my co-investigator and I will have access to them. We will not quote you without asking specific permission.

You should feel free at any time to ask me questions concerning the interview or the project or to decline to answer a question. Do you have any questions? OK, let’s get started.

It will help to define what we mean by public health and climate change up front.

We’re defining “public health” broadly to include the “upstream” or social, environmental and physical determinants of health, such as asthma from air pollution, housing conditions, education, income, access to healthy foods and transportation, *etc*. When we talk about “public health workers” or “public health organizations”, we are talking about people both inside and outside of government that are working on any aspect of trying to improve population and community health.

When we talk about the impacts of climate change on health, we’re talking about both direct impacts like extreme heat, indirect impacts like rising food prices from drought, and the health effects of various strategies to reduce greenhouse gas emissions such as decreased air pollution from higher fuel efficiency. Also, this project is focused more on the U.S. than on international issues, even though we understand that the global impacts are very significant.

(Note: * indicates questions you can skip if pressed for time.)

#### B2. General Level of Awareness/Concern about Climate Change and Health

First, can you tell me a little about your organization, and the type of work that you do with your organization.

Let’s start talking a bit about climate change. When you hear that term, what comes to mind? Probes:

- How does that story make you feel?
- Are there any stories or things you’ve read or heard that particularly resonate for you?
- What does this story or information make you feel?
- Why do you think it resonates for you? What is important about this?

Have your read or heard anything about the relationship between climate change and health or how climate change affects health? What have you heard?

* Are there particular things you’ve heard about climate change and health that are especially important to you? Probe: Are you concerned about a particular health endpoint, or something that impacts people’s health indirectly?

* How big of an issue do you think climate change is as a public health issue? Do you think it will be a bigger issue for public health in the future? Why/why not?

Do you ever talk about climate change with your co-workers, your staff, or your managers? Do you think they are aware of or concerned about climate change as a public health issue? Can you share any stories or examples that show their level of awareness or concern?

* We’re wondering what your clients or external stakeholders might think about the impacts of climate change on health. Can you give me some examples or stories that show their level of awareness or concern?

#### B3. Current Work Related to Climate Change and Health

Have you read or heard anything about climate change and health that you think may be related to the work you do every day and/or the mission of your program or organization?

Have you heard of the concept of co-benefits? Co-benefits are strategies to prevent further climatic changes that also have a beneficial effect on health and quality of life.

Would you say you are you currently involved in work related to climate change, health, or co-benefits, even in a very broad sense? *(Now follow one of two arrows below:)*

*If yes:* Please describe them. Do you make those connections explicit? If not, can you imagine making those connections more explicit? Would you be interested in further exploring some of these potential links between the work you are already doing and climate change?

- If not, why not?
- If yes, what links are you thinking about exploring more in your work?

*If no, prompt with:* Some of the ways we think climate change could be related to the work of your program include xxxxx… Is that something you would be interested in learning more about and exploring more in your work?

- If not, why not?
- If yes, explore how the connections could be made in their work.
- If no, explore why not.

#### B4. Opportunities and Barriers

* How comfortable would you be making the link to climate change when talking to your co-workers, community, or clients, and what would you need to support that? Explore comfort level and reasons.

What are some of the things that might help and support you and/or your organization become more interested or engaged with work related to climate change and health? Prompts:

- If no, explore why not.
- more information and understanding,
- time,
- resources,
- funding,
- leadership,
- peer support
- other supports?

Do you see other public health workers and public health organizations as having a role or responsibility in addressing climate change?

- funding,
- If so, what is that role?
- What are some of the things you think public health workers might do to address climate change?

Are there things that would make it challenging for you to become more involved in work related to climate change and health?

- Are there any larger “upstream” barriers that would prevent your organization or agency from becoming more involved in work related to climate change and health?

#### B5. Narratives about Public Health and Climate Change

How did you first get interested in public health? Probes:

- What do you find most rewarding about it?
- Can you tell me a story about an accomplishment or something you feel really good about in the work you are doing?
- Why does that story stick in your mind?
- How does that story make you feel?

What is the most disheartening aspect of your work? How do you deal with that?

Let’s start talking a bit about climate change. When you hear that term, what comes to mind? Probes:

- How does that story make you feel?
- Are there any stories or things you’ve read or heard that particularly resonate for you?
- What does this story or information make you feel?
- Why do you think it resonates for you? What is important about this?

#### B6. Networking and Closing

Are there people, publications, or organizations that you especially trust to give you good information that is relevant to your work?

Do you think you or your colleagues might be interested in attending trainings or discussions about climate change and health? If yes—webinar? Teleconference? In-person?

Would you be interested in receiving information from the PHI Center for Climate Change and Health by email?

Would you mind sharing the names of others who you think would be useful to interview on this subject? We are especially looking for other public health workers with some awareness or interest, and who may be able to increase their agency’s engagement. *(Get contact information)*

Do you have any questions for me/us? Is there anything else you’d like to share?

You’ve been so helpful; I really appreciate the time you’ve taken to talk with me today. I just want to confirm that we can list you in the acknowledgements section of our final whitepaper. Do you mind if we contact you in the future with any follow-up questions that may emerge? Thank you very much.

### C. “Non-Public Health” Interview Tool

Audience: Those who are not PH-identified, but who are doing climate change work (as self-defined), and who can facilitate bridging public health involvement.

Goals:

- To discover how PH can more successfully engage with the non-PH sector working on CC to increase effectiveness of action.
- To explore the extent to which people working on CC already see the health connections.

#### C1. Introduction

Thank you for agreeing to meet with me/us. We are working with the Public Health Institute Center for Public Health and Climate Change on a project funded by the Kresge Foundation. We see climate change as one of the greatest health and equity challenges of the 21st century, and we also see that many climate action strategies could have very positive impacts on health. The goal of this project is to find out how to make it easier for the public health community to work on climate change and strengthen partnerships between public health workers and others who are working on climate change. We also want to know how public health could help others working on climate change to bring a stronger health frame to their work. We are starting by speaking to people in public health, climate change, equity, planning, environmental justice, and other fields, to learn more about their work and get their ideas on what would be helpful.

Everything you tell me will be kept confidential, unless we specifically ask for your permission to quote you. I‘ll be taking notes, but only my co-investigator and I will have access to them. You should feel free at any time to ask me questions concerning the interview or the project, or to decline to answer a question. Do you have any questions? OK, let’s get started.

It will help to define what we mean by public health and climate change up front.

We’re defining “public health” broadly to include the “upstream” or social, environmental and physical determinants of health, such as asthma from air pollution, housing conditions, education, income, access to healthy foods and transportation, *etc*. When we talk about “public health workers” or “public health organizations”, we are talking about people both inside and outside of government that are working on any aspect of trying to improve population and community health.

When we talk about the impacts of climate change on health, we’re talking about both direct impacts like extreme heat, indirect impacts like rising food prices from drought, and the health effects of various strategies to reduce greenhouse gas emissions such as decreased air pollution from higher fuel efficiency. Also, this project is focused more on the US than on international issues, even though we understand that the global impacts are very significant.

#### C2. Relationship between Their Work and Public Health

First, can you tell me a little about the work you do and your organization does, especially that relates to climate change?

- What issues does your organization work on?
- Where does climate change fit in terms of your organization’s priorities?

Can you talk about how you see the links between climate and health and equity, either in terms of the impacts of climate change itself, or how climate mitigation and adaptation strategies might affect health or equity? Are you familiar with the concept of co-benefits? Co-benefits are strategies to prevent further climatic changes that also have a beneficial effect on health and quality of life.

*If necessary, prompt with:* Some of the ways we think health could be related to the work of your program include xxxxx… Is that something you would be interested in learning more about and exploring more in your work?

- What issues does your organization work on?
- If yes, explore how the connections could be made in their work.
- If no, explore why not.

#### C3. Framing

Do you have any thoughts about how the community or other stakeholders that you work with think about climate change? Do you have any stories or examples that show their awareness or concern?

How about how they view health? Do you have any stories or examples that show their awareness or concern?

Do you have any stories or examples of whether they are aware or concerned about the impacts of climate change on health?

In your experience, what are some of the challenges in talking to people in your community about climate change? Do you talk explicitly about climate change, or do you start from some different framing? If so, what/how? Do you ever/often talk about the links between climate change and health? Explore.

Why do you think the general public is not more concerned about climate change?

Do you see opportunities for getting people more engaged in climate change action and strategies through the use of a health frame? Are there any ways that using a health frame might either help or hinder your own efforts around climate change, or things we should be careful about as we try to engage more people in climate change using a health frame?

#### C4. Partnering with Public Health

Do you currently work with PH organizations – either government agencies like a local health department, or other organizations that work on issues like asthma or obesity? *(Now follow one of two arrows below.)*

*If yes:* Who have you worked with?

- Has that work with PH helped forward your agenda?
- What was successful about that partnership and what was challenging?
- How did working with that agency get started?
- Has that partnership changed anything in your or their work?
- What do you think you and others got out of having public health at the table?

*If no:* Would you like to be working with local public health groups more?

- What could support building that relationship?
- What are some of the barriers to you creating those partnerships?

Are there other (additional) things you’d like to see people in the PH community do that would help you in your work on climate change? *Probe*:

- What would you like to see your local PH groups or departments do?

Do you have any thoughts about what might help or support greater partnerships or collaboration on climate change between you and public health organizations?

Are there things that would make it challenging for you to bring health into your work more? Or that would be helpful for you in making the connections between the climate change work you do and health more explicit?

#### C5. Narratives about Public Health and Climate Change

What made you first get engaged in climate change work? Can you tell me what, when, or who made you start caring about it or thinking it was an important issue?

What do you find most rewarding about it?

Can you tell me a story about an accomplishment or something you feel really good about in the work you are doing?

- Why does that story stick in your mind?
- How does that story make you feel?

What is the most disheartening aspect of your work on climate change? How do you deal with that?

#### C6. Networking

Would you mind sharing the names of others who you think would be useful to interview on this subject? We are looking for people with some awareness or interest, and who may be able to increase the consideration of health in their climate change work. *(Get contact information)*

Are there people or publications that you especially trust to give you good information that is relevant to your work?

Would you be interested in receiving information from the PHI Center for Climate Change and Health by email?

Do you have any questions for me/us? Is there anything else you’d like to share?

You’ve been so helpful; I really appreciate the time you’ve taken to talk with me today. Do you mind if we contact you in the future with any follow-up questions that may emerge? I just want to confirm that we can list you in the acknowledgements section of our final whitepaper. Thank you very much.
